# Co-doped carbon materials synthesized with polymeric precursors as bifunctional electrocatalysts†

**DOI:** 10.1039/d0ra07100e

**Published:** 2020-09-30

**Authors:** Imran Karajagi, K. Ramya, P. C. Ghosh, A. Sarkar, N. Rajalakshmi

**Affiliations:** Centre for Fuel Cell Technology (CFCT), International Advanced Research Centre for Powder Metallurgy and New Materials (ARCI) 2^nd^ Floor, IIT-M Research Park, Block E, 6 Kanagam Road, Taramani Chennai – 600113 India ramya@arci.res.in; Centre for Research in Nanotechnology and Science (CRNTS), Indian Institute of Technology Bombay Powai Mumbai – 400076 India; Department of Energy Science and Engineering, Indian Institute of Technology Bombay Powai Mumbai – 400076 India; Department of Chemical Engineering, Indian Institute of Technology Bombay Powai Mumbai – 400076 India

## Abstract

The design of stable and high performance metal free bifunctional electrocatalysts is a necessity in alkaline zinc–air batteries for oxygen reduction and evolution reaction. In the present work co-doped carbon materials have been developed from polymeric precursors with abundant active sites to achieve bifunctional activity. A 3-dimensional microporous nitrogen–carbon (NC) and co-doped nitrogen–sulfur–carbon (NSC) and nitrogen–phosphorus–carbon (NPC) were synthesized using poly(2,5-benzimidazole) as an N containing precursor. The obtained sheet like structure shows outstanding ORR and OER performance in alkaline systems with excellent stability compared to Pt/C catalyst. The doped heteroatom in the carbon is expected to have redistributed the charge around heteroatom dopants lowering the ORR potential and modifying the oxygen chemisorption mode thereby weakening the O–O bonding and improving the ORR activity and overall catalytic performance. The bifunctional activity (Δ*E* = *E*_*j*=10_ − *E*_1/2_) of an air electrode for NPC, NSC, NC and Pt/C is 0.82 V, 0.87 V, 1.06 V and 1.03 V respectively, and the NPC value is smaller than most of the reported metal and non-metal based electrocatalysts. The ORR (from onset potential) and OER (10 mA cm^−2^) overpotential for NPC, NSC, and NC is (290 mV, 410 mV), (310 mV, 450 mV) and (340 mV, 600 mV) respectively. In the prepared catalyst the NPC exhibited higher ORR and OER activity (NPC > NSC > NC). The doping of P in NPC is found to have a great influence on the microstructure and therefore on the ORR and OER activity.

## Abstract 2

Metal free bifunctional catalysts based on co-doped carbon materials synthesized from polymeric precursors *via* a simple pyrolysis route with high cyclic stability and low polarization for Zn–air batteries.

## Graphical abstract

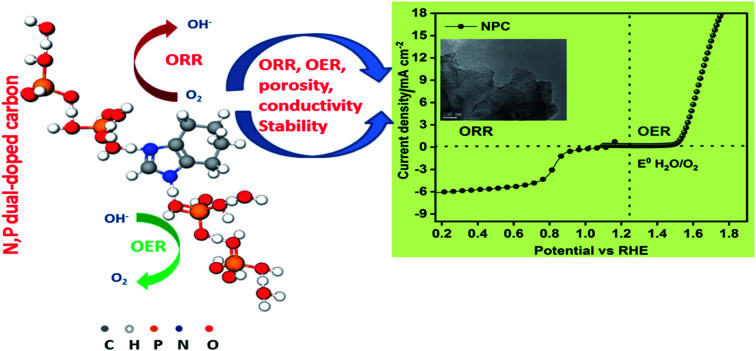

## Introduction

The rechargeable zinc–air battery has drawn more attention in energy storage applications owing to its high energy density, safety and cost effectiveness.^1,2^ However, large scale implementation of zinc–air batteries is restricted by many technical challenges of the oxygen evolution reaction (OER) and oxygen reduction reaction (ORR). The slower reaction kinetics of both the ORR and OER results in high overpotentials due to their complicated multi-electron processes and limits the output performance of zinc–air batteries.^3^ The exchange current densities for cathodic reactions are several orders lower than that of anodic reactions in zinc–air batteries.^4^ The OER is also equally critical to maintain voltage efficiency and recharge rate.^3^ Precious metal based catalysts such as platinum, iridium and ruthenium, *etc.* are normally used to overcome the overpotential problem for OER and ORR. But, their use for practical applications is limited by poor durability, high cost and scarcity.^5,6^ The air cathode design comprised of both the ORR and OER catalyst mixed together have been proposed with high complexity and system stability.^7^ With the above mentioned concerns, developing a cost effective and efficient single cathode catalyst with OER and ORR activity is desirable for secondary zinc–air batteries.

The reduction of oxygen at catalyst surface takes place *via* two-electron or four-electron reaction pathways.

1 O_2_ + H_2_O + 2e^−^ → HO_2_^−^ + OH^−^

2 O_2_ + 2H_2_O + 4e^−^ → 4OH^−^

However, the two electron pathway reduces the overall activity by producing an unfavourable superoxide.^8^ Competitions of two and four electron pathways are highly analogous with the selectivity of the catalyst, four electron pathways are highly desirable for achieving higher efficiency and energy density.

The OER in alkaline medium is a reverse activity of ORR and it is complex in nature. The reaction mechanism for OER process depends on the active sites and electrocatalyst materials. The four electron and proton transfer pathways are highly desirable to achieve lower overpotentials and higher performance. The air electrodes designed with porous materials loaded with transition metals like cobalt and manganese has emerged as non-precious metal (NPMCs) based catalyst.^9–13^ For example, Hao Wang *et al.* developed hollow porous ZnMnCoO_4_ microspheres with high oxygen reduction activity and a half-wave-potential that is only 50 mV lower than that of Pt/C counterpart and excellent durability in the alkaline solution.^14^ Trimetallic CoFeCr hydroxide electrocatalyst developed by Yibin Yang on carbon paper with low temperature (60 °C) hydrothermal approach displays significantly improved oxygen evolution activity in alkaline conditions with small overpotential of 260 mV at 10 mA cm^−2^ and Tafel slope of 40 mV dec^−1^.^15^ The unique IrO_2_ core-Ir shell nanostructure (IrO_2_@Ir) designed by Wenwu Zhong *et al.* exhibited low overpotentials of 250 mV at 10 mA cm^−2^ and Tafel slope of 45 mV dec^−1^ for OER.^16^ The synthesized rock salt type nanocomposites of Ni–Co oxides with different molar ratios supported on ordered mesoporous carbon (NiCo_2_O_3_@OMC) composite exhibited a superior OER activity and high stability with lower overpotential of 281 mV at 10 mA cm^−2^ and Tafel slope of 96.8 mV dec^−1^ in alkaline condition owing to the abundant active sites and oxygen vacancies due to nickel and cobalt oxides.^17^ Guangfu Liao *et al.* reported the Ag-based nanocomposites, including supported Ag nanocomposites and bimetallic Ag nanocomposites as highly efficient electrocatalysts.^18^ Yan-Jie Wang *et al.* developed strategies to produce doped carbon composite bifunctional electrocatalysts and studied the relationship between the bifunctional catalytic mechanisms of ORR/OER and the electronic structure/composition of catalysts using a combination of molecular/atomic modelling and experimental characterization, for further improvements in the ORR/OER catalytic performances.^19^

The problems associated with NPMCs are degradation of metal particle, agglomeration and leaching out of metal particles due to long term usage. This may be attributed to the intermediates like peroxides.^20^ Therefore, the research for replacement of metal based catalyst with comparable electrocatalytic activity has been extensively carried out. The low cost and wide distribution of carbon materials like carbon nanotubes, pyrolyzed polymers, covalent organic frameworks (COFs) and graphene have been studied extensively as OER and ORR catalyst.^21–28^ In addition, doping of single or more heteroatoms such as N, P, S and B to carbon materials are advantageous for electrocatalytic activity. It has been found that the multiple doping of heteroatoms can create strong synergistic coupling effect with unique electronic structure in the carbon framework.^29–31^ The catalytic activity of co-doped carbon is higher than single heteroatom doped carbon. The catalytic capabilities can also be tailored by altering the dopant types and doping sites and level of doping for various electrocatalytic reactions. For example, the onset potential and current density of catalyst can be influenced by the pyridine N and quaternary N, respectively, as per the studies carried out by Lai *et al.*^32^ The designed carbon nanotube arrays with nitrogen dopants exhibited much higher electrochemical performance than Pt/C in Gong's work.^33^ N, O doped carbon hydrogels exhibited better OER performance while, N, S or N, B co-doped graphene revealed much better ORR performance. Jiao *et al.* observed the lowest change in free energy for graphene doped with N and B heteroatoms and validated the experiments using DFT calculations.^34^ The change in spin and charge density distribution is altered by the multiple doping of heteroatoms. The spin density change holds the great importance as per DFT studies.^35^ The more significant spin density change is produced by co-doped materials than the mono-doped materials resulting in higher catalytic activity.^36^ Apart from the spin density change, the oxygen adsorption is also of equal importance. The multiple doping of heteroatoms alters the spin and charge density distribution concurrently. When doped with more electronegative nitrogen, it creates a positive charge on C and the neighbouring C can act as active site for oxygen reduction reaction. The less electronegative heteroatoms such as S, P and B serve as O_2_ adsorption sites themselves.^37^

Theoretically, the doped nitrogen heteroatom and the carbon atom in the matrix possess different electronegativity, bond length, atomic radii and induces defects and uneven distribution of electric charge states around the conjugated dopant sites. Thus, due to charge delocalization, carbon atoms become positively charged and enhance the ORR.^38,39^ Hence, with additional heteroatom dopants like S or P along with N in the carbon matrices, further modification in the electronic structure and creation of new active sites occurs and enhances the overall performance.

There are still many challenges to achieve controlled even distribution of doped species in heteroatom doped carbon systems. Heteroatoms are doped usually by thermal evaporation process involving heating of carbon materials with gaseous precursors of S and N (NH_3_, SO_2,_ pyridine, H_2_S, thiophene)^40,41^ or high temperature pyrolysis with some precursors like melamine and benzyl disulphide.^42^ However, there are limitations with these strategies like low doping efficiency even by using excessive N, S and P sources and use of very high temperature. In addition, precursors with higher N content are pyrolyzed to improve the doping efficiency of nitrogen in the carbon.^43–45^ Hence it is understood that precursor plays a major role in getting the required amount of dopants in the carbon matrix.

Our present synthesis method uses polybenzimidazole as N containing precursor to synthesise microporous carbon nanosheets as electrocatalyst for ORR and OER. Compared to other N containing precursors, polybenzimidazole displays many features such as high yield carbon and excellent structural tunability for fabricating desired nanostructures. Co-doped carbon materials can be easily prepared from polybenzimidazole as the –N present can interact with various inorganic and organic acids paving way for introduction of other heteroatoms like sulfur and phosphorous apart from nitrogen. Further polybenzimidazole contains two nitrogen atoms per monomer unit and can lead to high –N content in the product.

## Experimental sections

### Materials

Poly 2,5-benzimidazole (ABPBI) solution in methane sulfonic acid was purchased from Gharda polymers Ltd, 5 wt% Nafion was purchased from DuPont. Phosphoric acid (H_3_PO_4_), methanesulfonic acid (CH_4_SO_3_), potassium hydroxide (KOH), zinc oxide (ZnO) and ethanol were procured from Merck. ABPBI powder was obtained by precipitating the solution in water and washed with water until it is free of acids.

### Synthesis of catalysts

#### Material synthesis

To prepare nitrogen–sulfur and nitrogen–phosphorous dual-doped carbon, N rich polybenzimidazole (0.5 g) was soaked separately with methanesulphonic acid (5 ml) and phosphoric acid (5 ml), respectively at room temperature for 12 h.

**Scheme 1:**
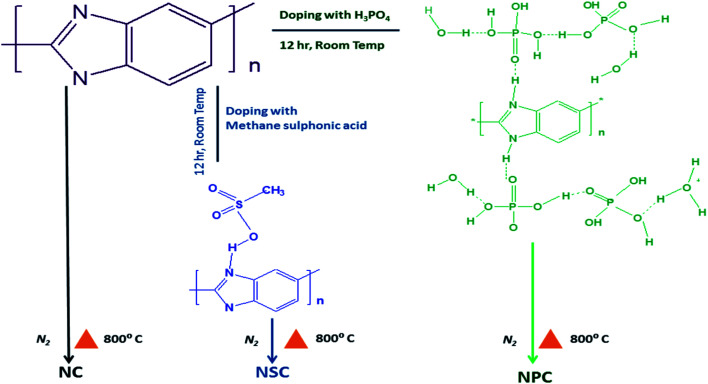
Synthesis scheme of sulfur and phosphorous co-doped metal free catalyst.

The treated polybenzimidazole was then transferred to alumina boat and was placed at high temperature furnace and purged with nitrogen at a flow rate of 50 ml min^−1^. The samples were carbonised at 800 °C for 6 hours at a rate of 10 °C min^−1^. The samples were cooled to room temperature under nitrogen atmosphere. The carbonised samples were washed thoroughly and dried. The samples were labelled as NSC and NPC. Polybenzimidazole without acid treatment was carbonised under same temperature to synthesis N-doped carbon and is labelled as NC. Scheme 1 illustrates the synthesis scheme.

#### Materials characterizations

The XRD analysis of the sample was performed using Rigaku D/Max-2500 diffractometer. The sample was scanned from 10 to 90 degree using Cu Kα radiation source (*λ* = 1.54 Å). The defects in the catalyst were studied by WiTech alpha R300 confocal Raman microscope using excitation source as Nd-YAG laser at 532 nm. The catalyst morphologies and elemental mappings were acquired on transmission electron microscopy (TEM, Technai G20) and field emission scanning electron microscopy (FESEM, Carl ZEISS Merlin Compact). Omicron Nano technology UK instrument was used to collect the X-ray photoelectron spectroscopy (XPS) data. Fitting was done by utilizing casaXPS. The rising edge of backscattered electrons in each high resolution spectrum was corrected with Shirley type background and was fitted with asymmetric Gaussian–Lorenzts (0–30%). The BET specific surface area of the as synthesized carbon based metal free electrocatalyst were calculated using Model Micromeritics ASAP 2000. The pore size distribution for the catalysts was calculated using Barret–Joyner–Halenda (BJH) model and BET isotherm.

#### Electrochemical characterization

The catalytic performance of the synthesized electrocatalyst was studied by a three electrode coupled electrochemical cell setup in alkaline 0.1 M KOH electrolyte solution using a bipotentiostat (Pine instrument attached with CH instrument). The working electrode was a slurry coated glassy carbon of disc area 0.245 cm^2^. The catalyst (1 mg) was dispersed in mixture of water (1 mL) and 5 wt% of Nafion (10 μL) solution and was sonicated well to obtain the catalyst slurry. Required amount of catalyst was coated on the working electrode. All measurements are recorded with a silver–silver chloride reference electrode and the potential values are converted to RHE. A graphite rod was used as a counter electrode. The electrochemical performance of the synthesized samples was assessed by scanning the potential of the catalyst coated working electrode from 0.2 V to 1.2 V *vs.* RHE and 1.0 V to 2 V *vs.* RHE for ORR and OER, respectively at a scan rate of 5 mV s^−1^ in O_2_ saturated 0.1 M KOH. The ORR measurements were recorded at different rotation per minute (rpm) and OER measurements were performed at 1600 rpm to eliminate the oxygen bubble formation at the electrode surface during the reaction process. The number (*n*) of electron and HO_2_^−^(%) percentage can be calculated by LSV polarization of the ORR process at different rotation using direct eqn (3) and (4):3
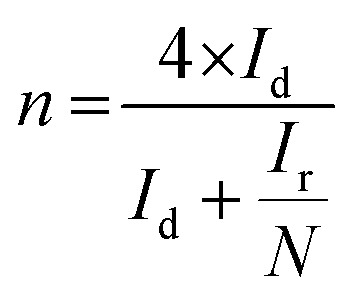
4
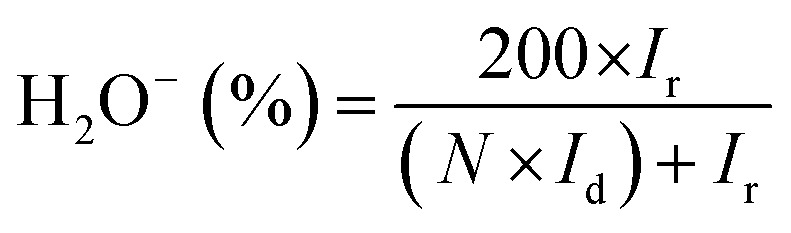
where, *I*_r_ and *I*_d_ is ring and disk current.

The power density (*P*) of zinc–air battery was calculated by

5 *P* = *I* × *V*

where *I* is the discharge current density and *V* is the corresponding voltage.

#### Zinc–air cell assembly and testing

The air electrode for secondary zinc–air battery was fabricated by following procedure:

The synthesized NPC/NSC catalyst was added to ethanol and dispersed well for half an hour. A 10 wt% of PTFE solution was mixed and the mixture was further stirred for half an hour. The prepared catalyst slurry of loading 12 mg cm^−2^ was coated on to SS mesh of 1 cm^2^ area and dried at 350 °C for 1 hour. The prepared electrode was used as air electrode at cathode and the anode electrode was prepared by electrodeposition of zinc on mild steel mesh. The zinc anode and air cathode were assembled into an acrylic setup with 25 ml of 6 M KOH+ 8 M ZnO as electrolyte. Battery cycling test were carried out using GCPL (Galvanostatic Cycling with Potential Limitation) method on Biologic battery cycler.

## Results and discussion

### Physical characterization

**Fig. 1:**
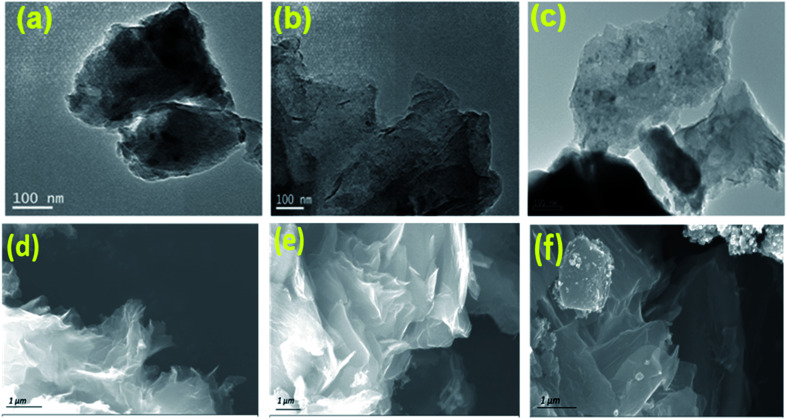
TEM image (a) NC (b) NSC (c) NPC. FESEM images of (d) NC (e) NSC (f) NPC.

The transmission electron microscopy (TEM) and field emission scanning electron microscopy (FE-SEM) was utilized to investigate the morphology of synthesized catalysts. The TEM image (Fig. 1a, b and c) of NC, NSC and NPC shows a number of *in situ* grown carbon layers embedded in hierarchical carbon network. NC, NSC and NPC maintain the sheet morphology and crinkles on the surface. Such carbon sheets are characteristics of small nanoscale graphitic units, assembled topologically disorderly with graphitization in short-range order as shown in Fig. 1d, e and f. The cracked and porous structure of these heteroatom doped carbon nanosheets creates many micro/mesopores which may serve as channels for the reactants to permeate through electrode, affording enhanced catalytic performance. Typical elemental mapping images of NC, NSC and NPC (Fig. S1†) illustrate the presence of C, N, S, P and O elements which are homogeneously distributed on carbon nanosheets.

**Fig. 2:**
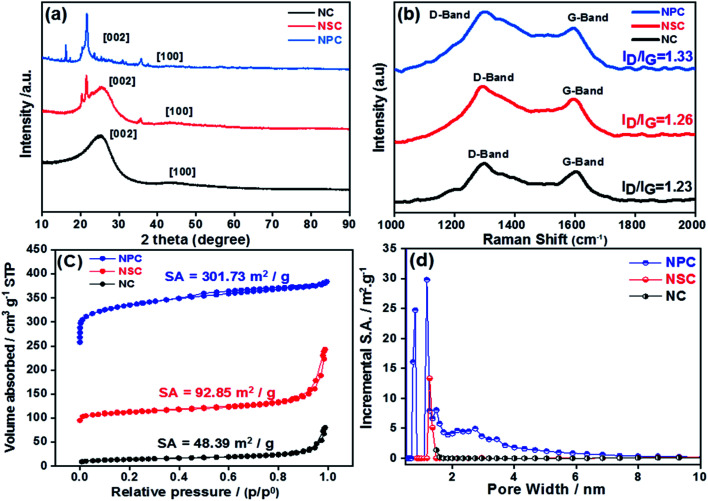
(a) XRD patterns of NC, NSC and NPC (b) Raman spectroscopy for NC, NSC and NPC catalyst (c) BET specific surface area of NC, NSC and NPC (d) pore size distribution of NC, NSC and NPC.

The structural properties of prepared catalysts were studied by X-ray diffraction (XRD). The formation of amorphous carbon with low crystallinity was confirmed through XRD patterns for all carbonised catalysts, which showed the broad peak at 25° (002) and 43° (100 and 101) from carbon as shown in Fig. 2a. The Raman spectroscopy was used to explore the graphitization degree for synthesized NC, NSC and NPC co-doped porous samples as shown in Fig. 2b. The existence of abundant defects in samples is indicated by the ratio of *I*_D_/*I*_G_ that can act as active sites. The D band originates from defects and G-band indicates the high degree of graphitization. The *I*_D_/*I*_G_ ratio of NC, NSC and NPC were determined as 1.23, 1.26 and 1.33, respectively. The higher *I*_D_/*I*_G_ ratio for NSC and NPC indicates that the defects in the carbon matrix increase after introduction of extra sulfur and phosphorous atoms which create the disordered moieties during the decomposition of polymeric structure.

The N_2_ adsorption–desorption isotherms were performed for the synthesized catalysts to determine the surface area and pore size distribution. All the catalysts displays the type-I isotherm, which attributes to the microporous materials as depicted in Fig. 2c and d. As summarized in Table 1 the BET specific surface area of NC is 48.39 m^2^ g^−1^, it increases to 92.85 m^2^ g^−1^ for NSC and further increases to 301.73 m^2^ g^−1^ for NPC. The average pore size and total pore volume follow the same trend. The pore size distribution suggests mainly the microporous structures (pore size less than 2 nm) with a small portion of mesopores (pore size between 2 and 50 nm). However, NPC has larger micropores and more pores belonging to mesopore. The micropores can expose more catalytic active sites, while mesopores are favourable for mass transfer process. Therefore, it is expected that NPC will display better catalytic activity and kinetics. The doping of S and P to carbon materials affects the surface area, pore size and pore volume of carbon framework. As the content of P-doping increases, the more defect sites will be induced in the carbon due to increase in surface area by distortion of hexagonal carbon matrix. The hierarchical porosity structures composed of microporous/mesoporous facilitate the fast mass transport with more catalytic active sites will results in improved electrochemical performance.^46^

**Table 1:** Physical properties of NC, NPC and NSC catalyst

Sample	Elemental composition at (%)					Raman *I*_D_/*I*_G_ ratio	BET surface area (m^2^ g^−1^)
	C	N	S	P	O		
NC	70.8	5.6			23.5	1.23	48.39
NSC	66.1	7.7	0.6		25.6	1.26	92.85
NPC	54.0	10.0		7.1	29.8	1.33	301.73

**Fig. 3:**
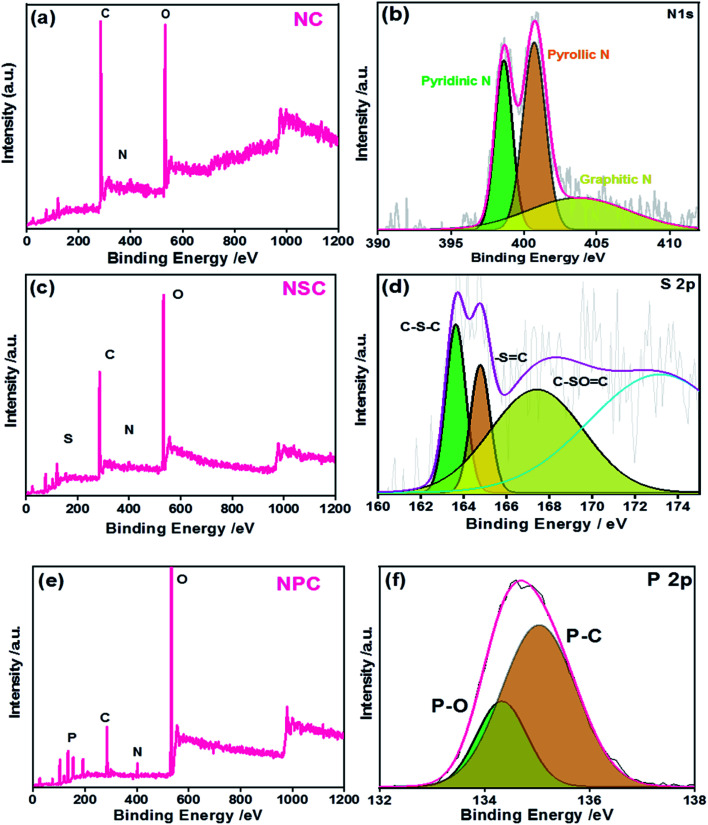
XPS (a) NC survey spectrum (b) N 1 s high resolution spectra (c) NSC survey spectrum (d) S 2p high resolution spectra (e) NPC survey spectrum (f) P 2p high resolution spectra.

The successful heteroatom doping and their chemical states were studied by XPS (Fig. 3). The presence of N, S and P were confirmed by their N 1s, S 2p and P 2p characteristic photoelectron peaks, in the samples. The survey spectrum is shown in Fig. 3a, c and e for NC, NSC and NPC.

The elemental composition of the NC sample was calculated to be 70.8, 5.6 and 23.5 atomic% for C, N and O, respectively. The spectrum of C 1s and N 1s were deconvoluted and the percentages of these species are given in Table 1. As shown in Fig. S2a,† the C 1s peak shows three major peaks attributed to C=C/C–C, C=N/C–N, and C=O functional groups at the binding energies of 284.6 eV, 285.8 eV and 287.9 eV, respectively. The N 1s spectrum of C–N yielded three peaks (Fig. 3b): at 398.5, 400.8 and 402.3 corresponding to pyridinic N (N=C), pyrolic N (C–N–C) and graphitic N(C–N^+^–C), respectively. The occurrence of N=C, C–N–C and C–N^+^–C or graphitic nitrogen proves the presence of nitrogen dopant in the carbon framework during pyrolysis process.

In the case of NSC sample, S 2p peak was observed in addition to the C 1s and N 1s. The deconvoluted spectra of C 1s and N 1s were similar to the NC sample (Fig. S2b†). The N 1s and S 2p spectrum was deconvoluted to investigate the bonding configuration in the NSC sample. The deconvoluted S 2p spectrum (Fig. 3d) yielded with three peaks at 163.7 eV, 164.9 eV and 167.9 eV, corresponding to S 2p_3/2_ (C–S_*x*_–C), S 2p1/2 (–S=C–) and C–SO_*x*_–C (*x* = 2, 3, 4).

The successful co-doping of heteroatoms was confirmed by the XPS survey spectrum of NPC showing the presence of P 2p and N 1s peaks along with C 1s. The graphitic N and pyridinic N account for the majority of N species in NPC, which are generally considered beneficial for ORR.^47^ The high resolution spectra of P 2p (Fig. 3f) showed the existence of P–O and P–C bonding at the binding energies of 134.2 eV and 135 eV, respectively, indicating the successful incorporation of P atoms in the carbon framework. The developments of P–O/C bonds are beneficial for ORR because it creates electron-deficiency at the C atom.

The total doping concentration of N and P in NPC sample is higher than the total doping concentration of N and S in the NSC sample. This could be attributed to higher doping of phosphoric acid with polybenzimidazole compared to the doping of methane sulphonic acid. The large atomic radius of P creates many distortions and defects leading to distribution of delocalized electrons over the surface. The deconvoluted spectra of C 1s and N 1s of NPC showed the same components as observed in NC and NSC samples. However, the C–C peak is positively shifted to 284.4 eV (Fig. S2c†). The positive shift in binding energy of the C–C is due to increased charge transfer from carbon atoms to heteroatoms. This will reduce the adsorbed oxygen efficiently by increase in electron transfer in the presence of heteroatoms.

### Electrochemical characterization

**Fig. 4:**
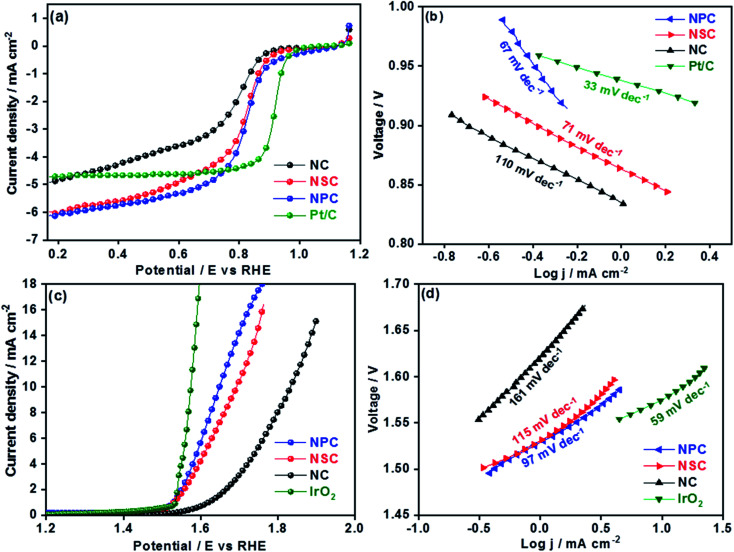
(a) Cathodic linear sweep voltammetry curves of NSC, NPC, NC and Pt/C at 1600 rpm in 0.1 M KOH oxygen saturated solution (b) Tafel slopes from the cathodic linear sweep voltammetry curves (c) anodic linear sweep voltammetry curves of NC, NSC, NCP and IrO_2_ catalysts at 1600 rpm. (d) Tafel slopes from obtained anodic linear sweep voltammetry curves.

The electrochemical performances of synthesized catalysts were explored by RRDE coupled with electrochemical workstation. The cyclic voltammograms of all the catalysts were performed under oxygen and nitrogen saturated 0.1 M KOH. The CV curves in N_2_ for all the catalyst shows the rectangular shape pure capacitive behaviour. The voltammograms in O_2_ saturated KOH showed the significant ORR peaks for all the catalysts at 0.7 V as shown in the Fig. S3.† Among all the catalysts, nitrogen and phosphorous co-doped NPC catalyst showed the prominent cathodic peak than NSC and NC catalysts suggesting the higher oxygen reduction activity. The dual doping of carbon materials with N–S or N–P can further increase the ORR activity by modulating the surface polarities and electronic properties. The linear scan voltammograms (LSV) studies for all the catalysts were performed at different rotation rates (400–2025 rpm) (Fig. S4†). The obtained linear sweep curves in 0.1 M KOH (O_2_-saturated) at 1600 rpm is depicted in Fig. 4a. The ORR onset potential for NC, NSC and NPC were found to be 0.89 V, 0.92 V and 0.94 V, respectively and their corresponding *E*_1/2_ potentials were found to be 0.77 V, 0.81 V and 0.82 V. These values show that the NSC and NPC catalysts outperform many of the reported metal and metal-free electrocatalysts and are comparable to precious metal based electrocatalysts (Table 2). Furthermore, the limiting current densities are much higher than that of Pt/C (4.8 mA cm^−2^). As observed, the co-doped catalyst NPC and NSC has the highest electrocatalytic activity in terms of limiting current and onset potential compared to NC catalyst. This highlights the importance of N, P and N, S co-doping and microporous structure for ORR.

**Table 2:** Electrochemical performance comparison for NC, NSC, NPC and other metal and metal free electrocatalysts

Electrocatalysts	OER			ORR			Δ*E*	Ref.
	*E* (V) onset	*E* @ 10 mA cm^−2^	Tafel (mV dec^−1^)	*E* (V) onset	*E*_1/2_ (V)	Tafel (mV dec^−1^)		
NC	1.57	1.83	161	0.89	0.77	110	1.06	**This work**
NSC	1.51	1.68	115	0.92	0.81	71	0.87	**This work**
NPC	1.50	1.64	97	0.94	0.82	67	0.82	**This work**
La_0.5_Ca_0.5_CoO_3−*δ*_		1.66	172	0.85	0.52	95	1.14	62
FeCo-NCNFs-800		1.68	105	0.9	0.81	60	0.86	63
Co_9−*x*_Ni_*x*_S_8_/NC		1.65	114	0.91	0.86	74	0.788	64
CeO_2_/Co@NCH		1.68	129	0.85	0.77		0.93	65
TA-NiFe@NCNT		1.54	78	0.93	0.81		0.79	66
N, P carbon paper	1.53	1.63	61.6	0.94	0.67	122	0.96	67
N–graphene/CNT		1.63	83	0.88			1.10	68
CaMn_4_O_*x*_							0.93	69
Mn_*x*_O_*y*_/N–carbon							1.04	70
NPCN-900				0.92	0.78	143		71
N,P-MC					0.84	58		72
Nitrogen-doped graphene/CNT composite				0.72	0.58		1	73
CPCTNB@CNTs				0.94	0.85	70		74
BNPC-1100	1.38		201	0.89	0.79	80		75
N, S–CN		1.64	53	0.91	0.76	89	0.88	76

The S-doping in the NSC sample plays major role in the enhancement of catalytic activity by providing more active sites for ORR and favour electrolyte ions migration and electron transport owing to its “electron spin density”. The higher the loading of heteroatoms the lower is the overpotentials of ORR. The experimental results show that the dual doping of the N, S and N, P enhances the ORR activity by creating large number of active sites and lowering the overpotentials. The higher surface area and the interconnecting ordered larger micropores in combination with mesopores forms an open network, providing a fast pathway to the active sites, through which the reactants and the products can be readily transported to and away from the active sites, avoiding mass transport limitations.^48,49^ The difference in ORR catalytic kinetics of metal-free catalysts was investigated by obtained Tafel slope from the polarization curves. The NPC catalyst displayed a lower Tafel slope (67 mV dec^−1^) than NSC (71 mV dec^−1^), NC (110 mV dec^−1^) and is comparable to Pt/C (33 mV dec^−1^) (Fig. 4b). The smaller Tafel slope of NPC indicates the higher intrinsic ORR activity and faster reaction kinetics.^50,51^

The overall catalytic activity of the samples was influenced by the performance of individual catalytic site and their density. In this work, the improved ORR activity of NPC sample compared to NC and NSC samples is due to charged catalytic sites of P+ or/and asymmetric spin density in the carbon atoms.^52,53^ The increase in amount of P doping increases the specific surface area and subsequently the density of catalytic sites.

The OER is equally important in the charging process of secondary Zn–air batteries. The OER performance of NC, NSC and NPC is evaluated and compared with state-of-art precious metal electrocatalyst (IrO_2_). The OER properties of catalysts were evaluated by LSV at a rotation speed of 1600 rpm. The improved OER catalytic activity for NPC was reflected by its higher current and earlier onset potential than those of NSC and NC as shown in Fig. 4c. As a reference, the OER for state of art IrO_2_ catalyst was performed for comparison. At a current density of 10 mA cm^−2^ the observed potential values were 1.64 V, 1.68 V, 1.83 V and 1.57 V for NPC, NSC, NC and IrO_2_, respectively. The measured OER kinetics shows that the NPC catalyst had lower Tafel slope of 97 mV dec^−1^, compared to NSC (115 mV dec^−1^), NC (161 mV dec^−1^) and is comparable to IrO_2_ (59 mV dec^−1^) (Fig. 4d). The reaction rate and kinetics of measured OER indicates the higher activity for NPC catalyst followed by NSC and NC. The NSC and NPC catalysts demonstrated better or comparable OER performance to many reported metal and metal-free based OER electrocatalysts (Table 2).

**Fig. 5:**
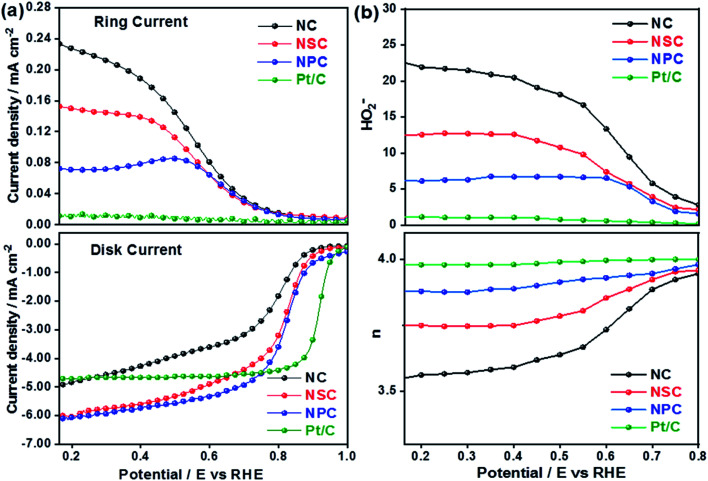
(a) Disk and Ring current densities (b) obtained number of electrons and percentage of HO_2_^−^ yield.

The superior OER catalytic activity of NPC may arise from its chemical structure, high surface area and porosity. The initiation of critical OER process involves the adsorption of OH^−^ and H_2_O, and the carbon atoms with positive charge around the heteroatom dopants such as N and P helps to promote the electron transfer by providing sufficient sites between reactants and catalysts.^54^ Further studies are required to understand the detailed OER mechanism in dual-doped (N and P) electrocatalysts. RRDE measurements were performed to calculate the number of electron transfer and peroxide yield (Fig. 5a and b) from disk and ring current densities for synthesized catalysts. The electrons transfer per oxygen molecule (*n*) was measured using eqn (3). The corresponding *n* values for NC, NSC, NPC, and Pt/C were 3.55, 3.74, 3.87, and 3.97, respectively. The HO_2_^−^ percentage was calculated using eqn (4) and found to be 22.6%, 12.5%, 6.0%, and 1.4% for NC, NSC, NPC, and Pt/C, respectively.

**Fig. 6:**
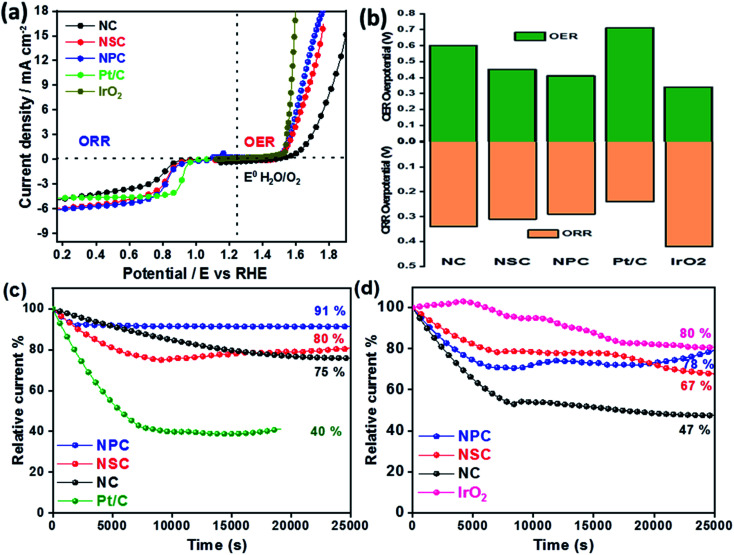
(a) Overall LSV curve for bifunctional ORR and OER activity. (b) Overpotentials of NC, NSC and NPC catalyst *vs.* RHE. (c) Chronoamperometric responses of NC, NSC, NPC and 20% Pt/C at 0.75 V *vs.* RHE(d) Chronoamperometric responses of NC, NSC, NPC and 20% Pt/C at 1.5 V *vs.* RHE.

The difference in the potential of OER and ORR (Δ*E* = *E_j_*_=10, OER_ − *E*_1/2, ORR_) metrics evaluates the catalytic activity of air electrode (Fig. 6a). The smaller the Δ*E* value of catalyst, higher is the bifunctional activity for the potential application in zinc–air battery as electrocatalyst. The Δ*E* value obtained for the NPC was 0.82 V, which is much more superior to NSC (0.87 V), NC (1.06 V). Electrochemical performance comparison of benchmark and various recent bifunctional catalysts are tabulated (Table 2), further confirming of superior ORR and OER performance of NPC catalyst.

The overpotentials of the synthesized catalyst for ORR (from onset potentials) and OER (@10 mA cm^−2^) was tabulated (Fig. 6b). The overpotentials for NC, NSC, NPC, Pt/C and IrO_2_ are (0.34 V, 0.60 V), (0.31 V, 0.45 V), (0.29 V, 0.41 V), (0.24 V, 0.71 V) and (0.42 V, 0.34 V), respectively.

The stability of synthesized catalyst for ORR was assessed by chronoamperometric measurements as shown in Fig. 6c. The catalyst NPC, NSC, NC maintains 91%, 80%, 75%, respectively of its initial current density after continuous operation for 7 h at 0.75 V compared to benchmark 20 wt% Pt/C (40%). The degradation of relative current of Pt/C catalyst is due to loss in the active surface area under a constant applied potential which is avoided in case of metal-free catalyst. Fig. 6d shows that the NPC, NSC and NC retains 78%, 67% and 47%, respectively of its initial current density after continuous operation for 7 h at 1.5 V compared to benchmark IrO_2_ (80%). The above results demonstrate the better stability of synthesized bifunctional electrocatalyst for long-term usage.

The improved exceptional bifunctional activity of NPC and NSC is due to the presence of multiple heteroatom dopants along with its unique porous structure and superior charge-transfer ability. The dual doping of S or P along with N creates more catalytically active centres due to positively charged S and P atoms and the high positive charge density created by electron accepting N species around sp^2^-bonded C atoms.^55–58^ The N and P or N and S co-doping also induces asymmetrical charge and spin density and increase the carbon active sites due to the synergistic effect.^59^ In addition, the excess of O species consequently accelerates the catalytic processes by providing the highly hydrophilic characteristic for more accessible catalytic surfaces.^60,61^

**Fig. 7:**
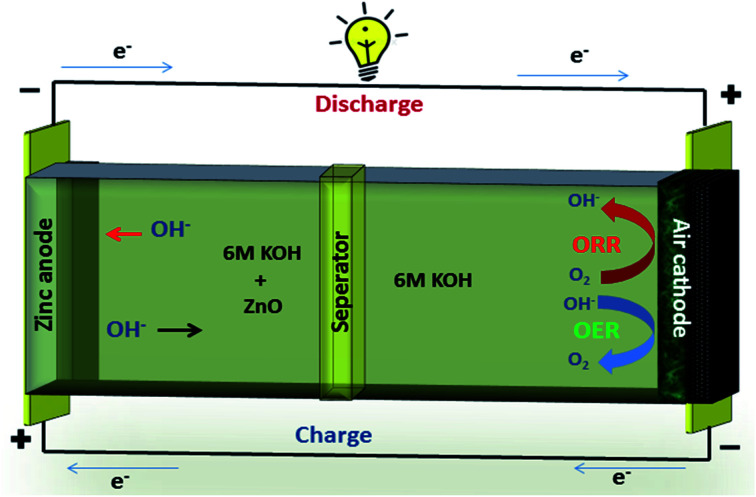
Zinc–air schematic.

A typical zinc–air battery consists of a Zn anode, air cathode, a separator and electrolyte as shown in Fig. 7. A secondary zinc–air battery uses aqueous 6 M KOH as electrolyte. Additives like Zn(Ac)_2_ or ZnO are mixed into the electrolyte to facilitate the reversible conversion of Zn anode in rechargeable Zn–air batteries.

Electrodeposited zinc or plate forms anode of Zn–air battery.^77^ The catalyst coated on SS mesh forms the cathode of Zn–air battery. The air electrode consists of catalyst and highly-hydrophobic PTFE for oxygen permeability and prevention of blockage of air passage by aqueous electrolyte.^78^

The electrochemical reactions of zinc–air battery as follows6
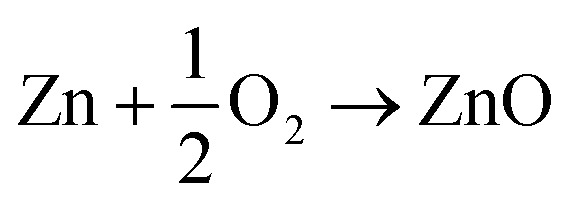


The zinc electrode generates Zn(OH_4_)^2−^ as intermediate by reacting with OH^−^ ions during discharge, and this further decomposes to ZnO.^79^

7 2Zn + 4OH^−^ → 2ZnO + 2H_2_O + 4e^−^

**Fig. 8:**
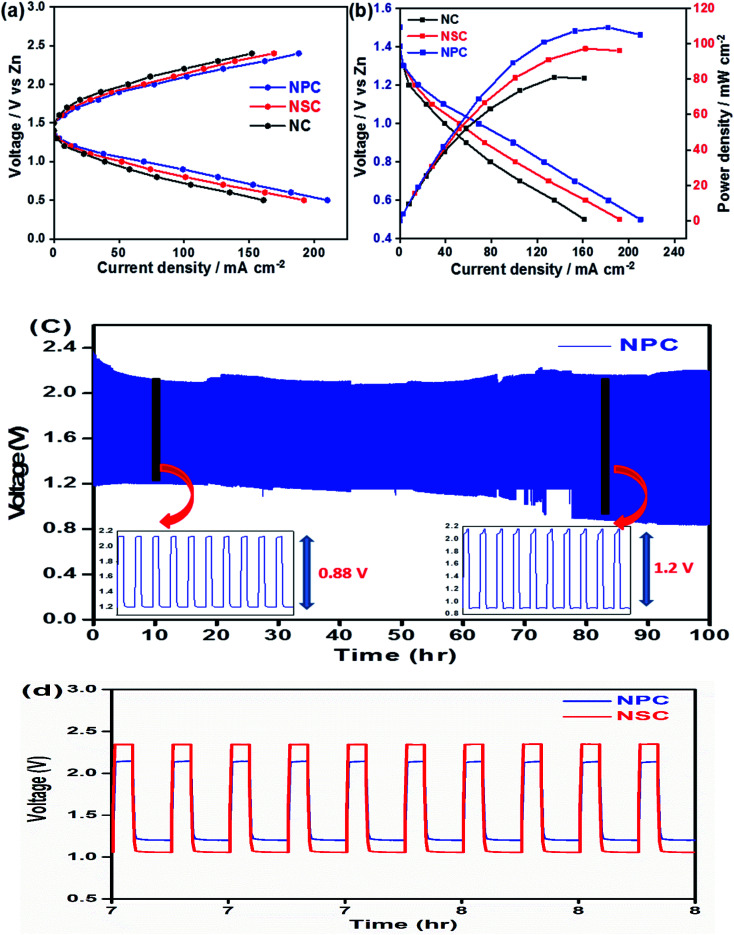
(a) Galvanostatic polarization curves for NPC, NSC and NC. (b) Resulting Power density and polarization curve for NPC, NSC and NC catalyst at air cathode. (c) Charge discharge cycling of NPC with 10 mA cm^−2^ of discharge current density. (d) Voltage polarization comparison between NSC and NPC at 100^th^ cycle.

The use of bifunctional electrocatalysts in the application of rechargeable zinc–air batteries is highly desirable. We further investigated the probability of using NPC and NSC electrocatalysts in the assembled rechargeable zinc–air battery. The open-circuit potential (OCP) for NPC catalyst is as high as ∼1.43 V in Zn–air battery, indicating the good catalytic activity of NPC. Fig. 8a and b depicts the discharge and charge voltage profiles and power density curves for NPC, NSC and NC as air electrodes. The catalyst NPC exhibited high discharge current density of ∼211 mA cm^−2^ compared to NSC and NC of 192 mA cm^−2^ and 161 mA cm^−2^, respectively at 0.5 V. The NPC cathode achieved a maximum current density of 188 mA cm^−2^ at 2.4 V which is higher than NSC and NC of 168 mA cm^−2^ and 152 mA cm^−2^, respectively during charging process. The maximum peak power density for NPC, NSC and NC was found to be 110 mW cm^−2^, 97 mW cm^−2^ and 81 mW cm^−2^, respectively (Fig. 8b). This clearly suggests the higher performance of synthesised catalyst compared to previously reported values.^80–82^ The highest peak power density and charge/discharge current densities are summarized in Table 3.

**Table 3:** Full cell performance comparison for synthesized catalysts and Pt/C

Sl number	Electrocatalyst	Max discharge current density (mA cm^−2^)	Max charge current density (mA cm^−2^)	Max power density (mW cm^−2^)
1	NPC	211	188	110
2	NSC	192	168	97
3	NC	160	152	81
4	Pt/C	280		139

The Fig. 8c shows the cycling performance of NPC as air electrode in zinc–air battery at 10 mA cm^−2^ of charge and discharge current density for 900 cycles. The voltage polarization for charge–discharge of NPC catalyst was proposed over 900 cycles. The initial voltages for discharge and charge were found to be ∼1.22 V and ∼2.1 V and these values were steady for initial cycles and the corresponding voltaic efficiency (discharge voltage/charge voltage) was found to be 58%. However, after 800 cycles the voltage gap for charge and discharge increased to ∼2.2 V and ∼0.9 V. Also, the obtained voltaic efficiency after 800 cycles was dropped to 40%. The cycling performance of NPC was compared with NSC as shown in Fig. 8d. The increase in the round trip overpotential for synthesized NPC and NSC catalyst 100 to 800 cycles was found to be 0.88 V to 1.2 V and 1.3 V to 1.75 V, respectively. The lowest change in voltage polarization and round trip peak overpotential of NPC catalyst suggests the excellent cell performance in zinc–air battery. The achieved full cell performances of synthesized catalysts were compared to other metal and metal-free based electrocatalysts reported in literature (Table S1†).

Conclusively, the synergistic effect of N and P doping has improved overall performance of the NPC catalyst. The change in C–P bond angle and bond length induces abundant edge defects in the carbon network which acts as active sites for oxygen adsorption. The atomic spin densities and charge densities play major role in the oxygen reduction activity of carbon materials. The N doping destroys the electric neutrality in the carbon matrix. Hence, the non-uniform charge density and enlarged spin density distribution of C atoms enhance the adsorption and reduction of O_2_. Further P doping introduces the asymmetric spin density with their available electron pair in the adjacent carbon atoms. The amount of active sites is increased further in the N, P co-doped pyrolytic carbon due to the presence of P–C bonds. Additionally, the large surface area 3D microporous graphene-like carbon network structure facilitates electrolyte and reactant diffusion to enhance the ORR and OER.

In addition to the high electron conductivity of carbon based materials compared to metal oxide based catalysts, they are also cost effective in mass manufacturing and environmental friendly due to the absence of metal ions getting released to the atmosphere.

The stability and composition of synthesized NPC catalyst were further studied after the operation of over 900 cycles using XRD and XPS techniques. Fig. S5† shows the XRD patterns of fresh and cycled electrode. It can be evident that the existence of peak at 23° in cycled electrode was consistent with the fresh counterparts. Fig. S6† shows the survey spectrum of NPC catalyst of fresh and cycled. The high resolution peaks of N 1 s and P 2p of cycled electrode is similar to the fresh electrode. These spent catalyst characterizations confirms the stability and composition of prepared catalyst.

## Conclusion

In summary, we developed N, N–S and N–P co-doped carbon materials synthesized with polymeric precursors *via* simple pyrolysis route. The synthesized metal-free catalyst with high porosity helps in the movement of gaseous products and lowers the transport resistance. Owing to the dual-doped system with unique structure and synergistic effect, outstanding electrocatalytic activity and superior stability for oxygen reduction and oxygen evolution reactions compared to many reported transition metal and metal-free based electrocatalysts was observed. The zinc–air battery using NPC as electrocatalyst showed the lower round trip peak potential and excellent stability over 900 cycles of continuous operation. We believe this work will offer a new aspect to design an electrocatalysts for the large scale application in the rechargeable zinc–air batteries.

## Conflicts of interest

There are no conflicts of interest.

## Supplementary Material

RA-010-D0RA07100E-s001
