# Supplementary material for: Co-doped carbon materials synthesized with polymeric precursors as bifunctional electrocatalysts
Source: RSC Adv. 2020 Sep 30;10(59):35966–78. doi: 10.1039/d0ra07100e (PMC9056983; doi:10.1039/d0ra07100e)
Supplement: RA-010-D0RA07100E-s001 [file RA-010-D0RA07100E-s001.pdf]

Electronic Supplementary Material (ESI) for RSC Advances.
This journal is © The Royal Society of Chemistry 2020

Co-doped carbon materials synthesized with polymeric precursors as bifunctional electrocatalyst

Imran Karajagi[a, b], K. Ramya[a,*], P.C.Ghosh[c], A. Sarkar[d], N.Rajalakshmi[a]

[a]Centre for Fuel Cell Technology (CFCT), International Advanced Research Centre for Powder Metallurgy and New Materials (ARCI), 2nd Floor, IIT-M Research Park, Phase-1, 6, Kanagam Road, Taramani, Chennai - 600113, India.

[b]Centre for Research in Nanotechnology and Science (CRNTS), Indian institute of Technology Bombay, Powai, Mumbai – 400076, India.

[c]Department of Energy Science and Engineering, Indian institute of Technology Bombay, Powai, Mumbai – 400076, India.

[d]Department of Chemical Engineering, Indian institute of Technology Bombay, Powai, Mumbai – 400076, India.

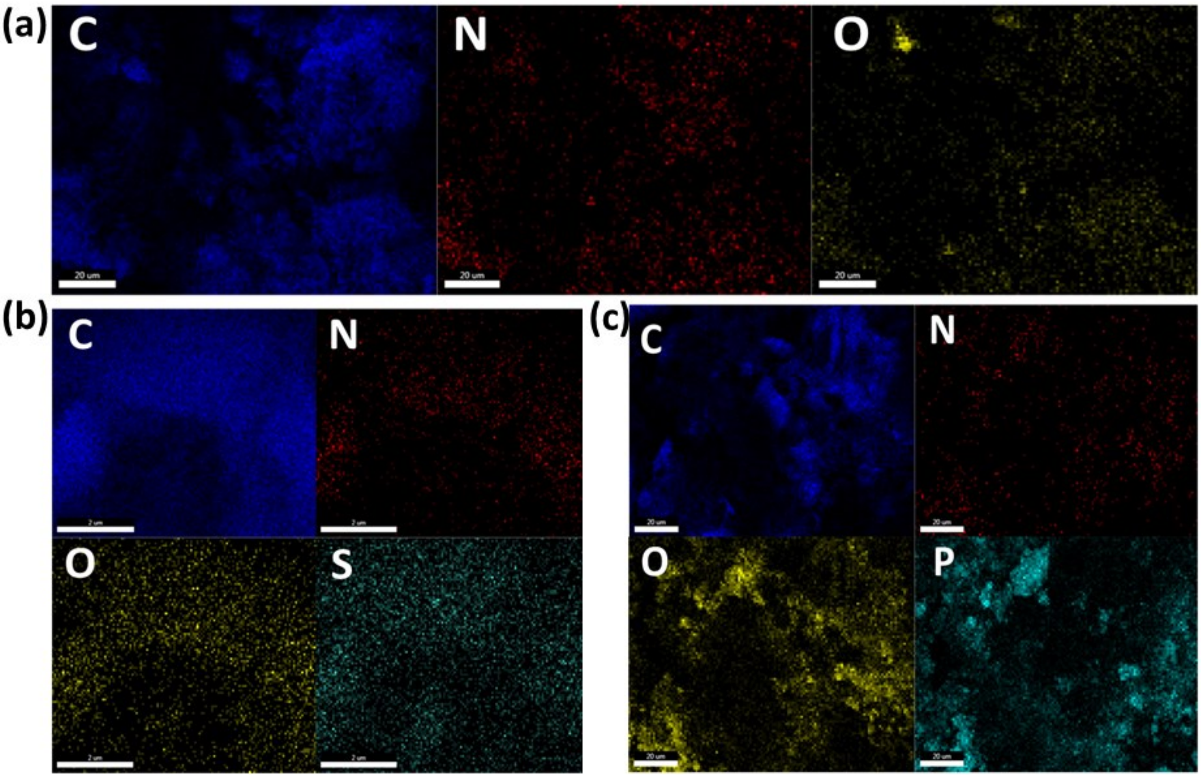

Figure S1: FESEM elemental mapping of C, N, O, S and P in (a) NC, (b) NSC and (c) NPC catalyst.

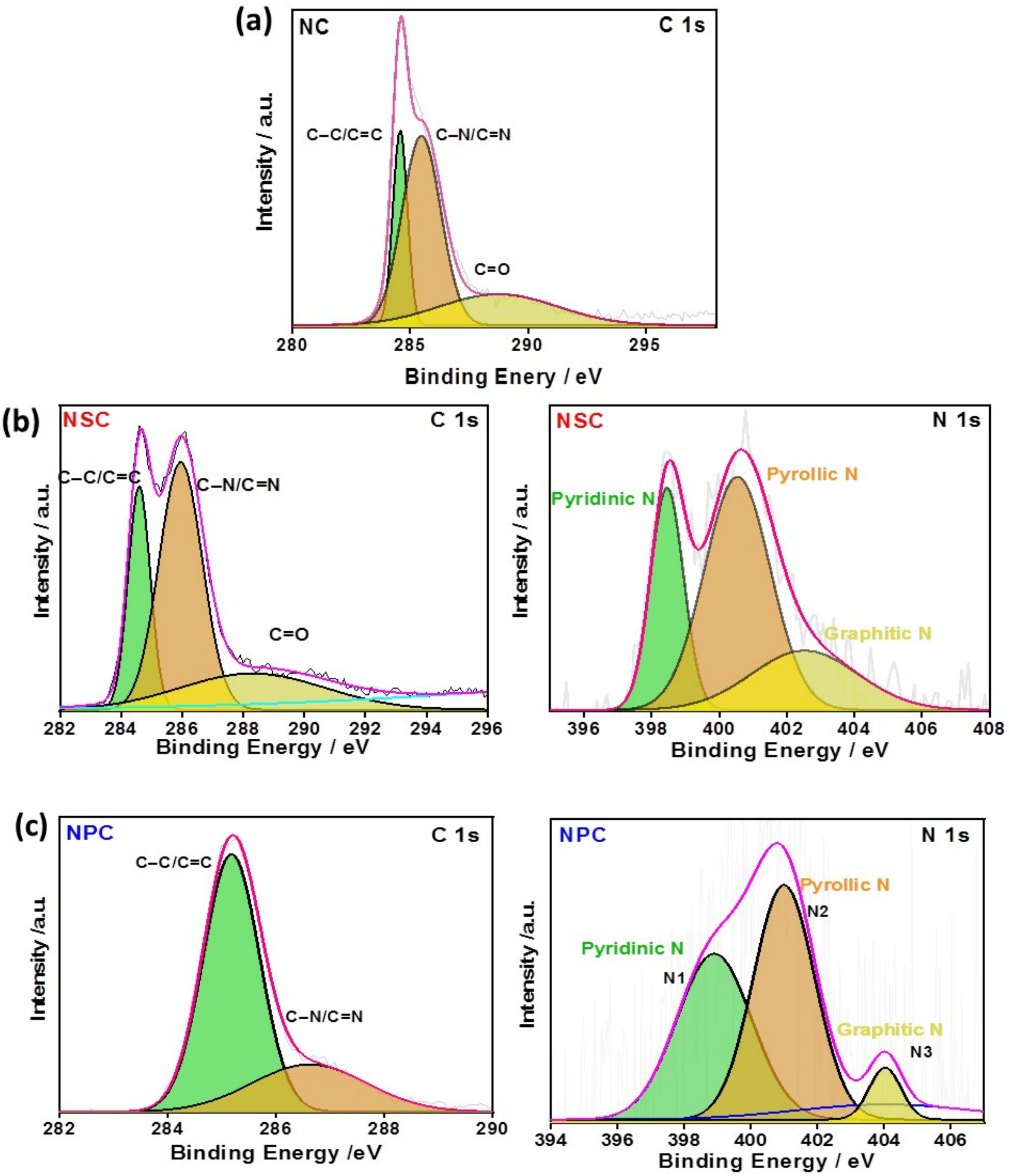

Figure S2. XPS (a) C 1s high resolution spectra of NC (b) C 1s and N 1s high resolution spectrum of NSC (c) C 1s and N 1s high resolution spectrum of NPC.

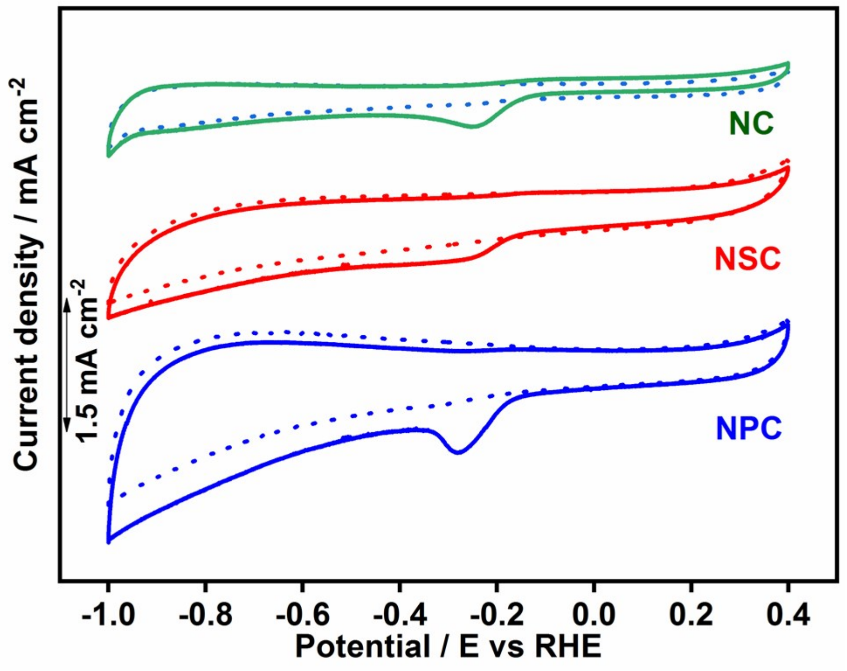

Figure S3. Cyclic voltammetry curves of NSC, NPC, NC

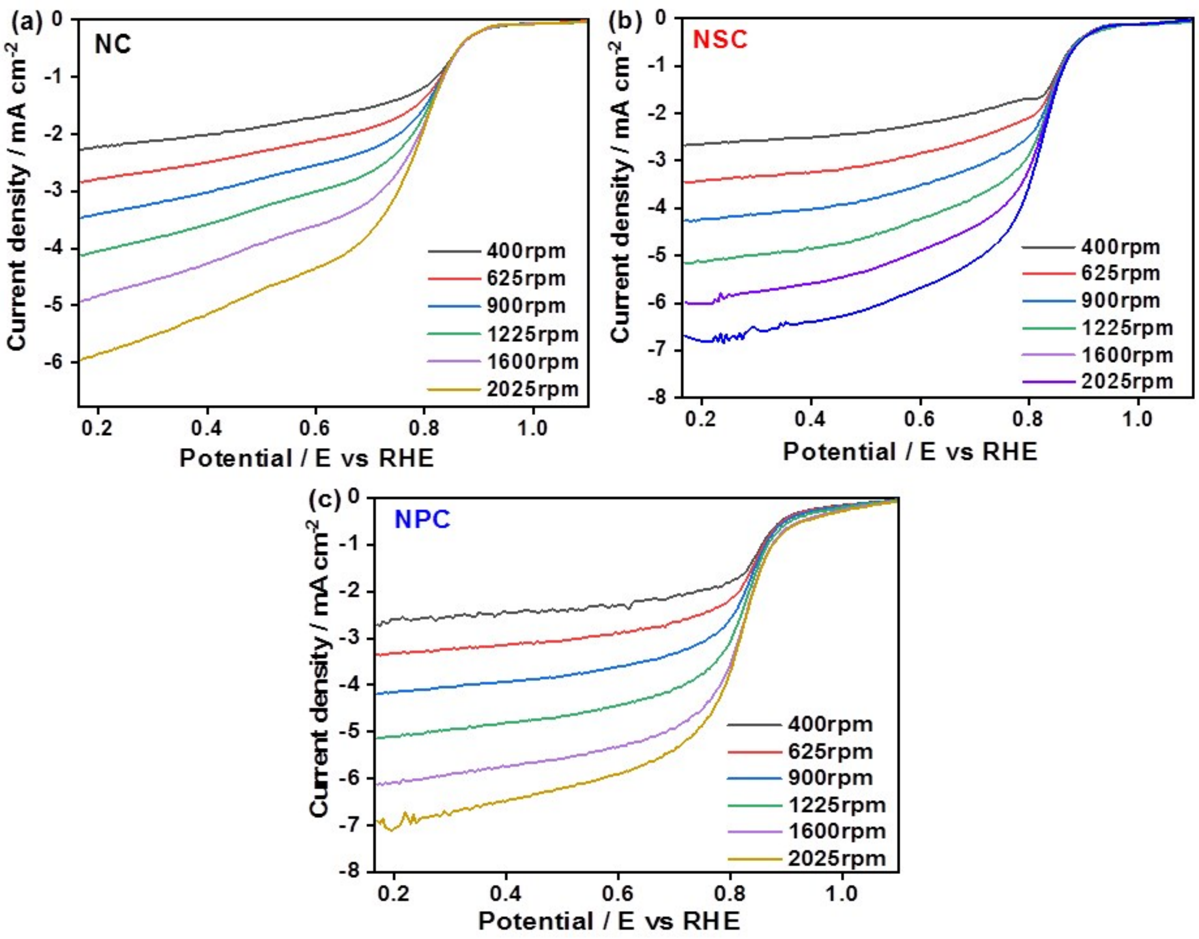

Figure S4. Cathodic linear sweep voltammetry curves of (a) NC, (b) NSC and (c) NPC at various rotations.

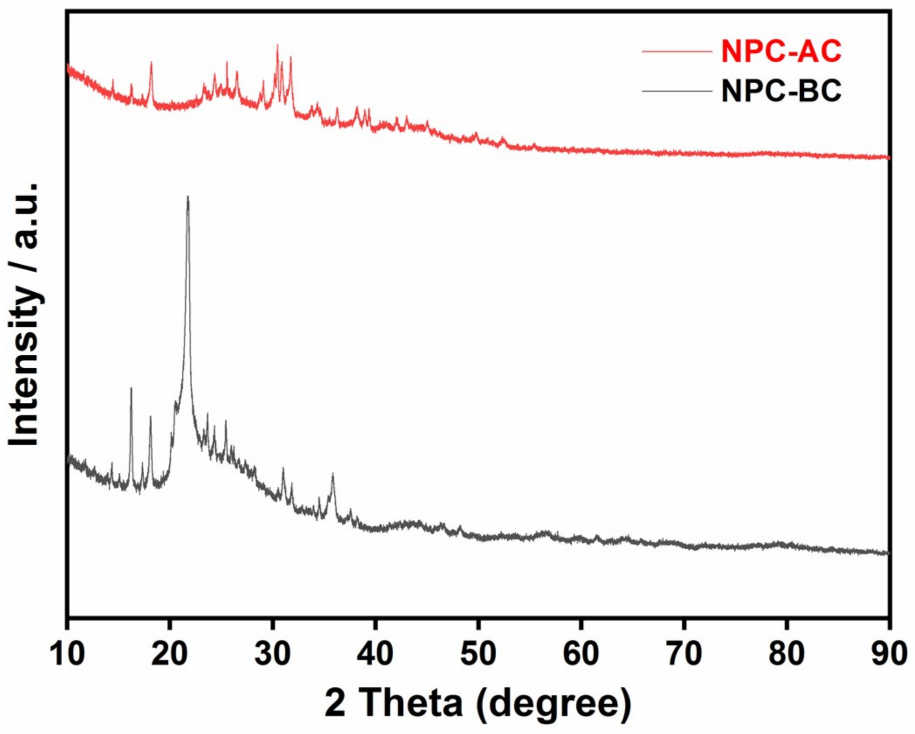

Figure S5. XRD of NPC before and after cycling.

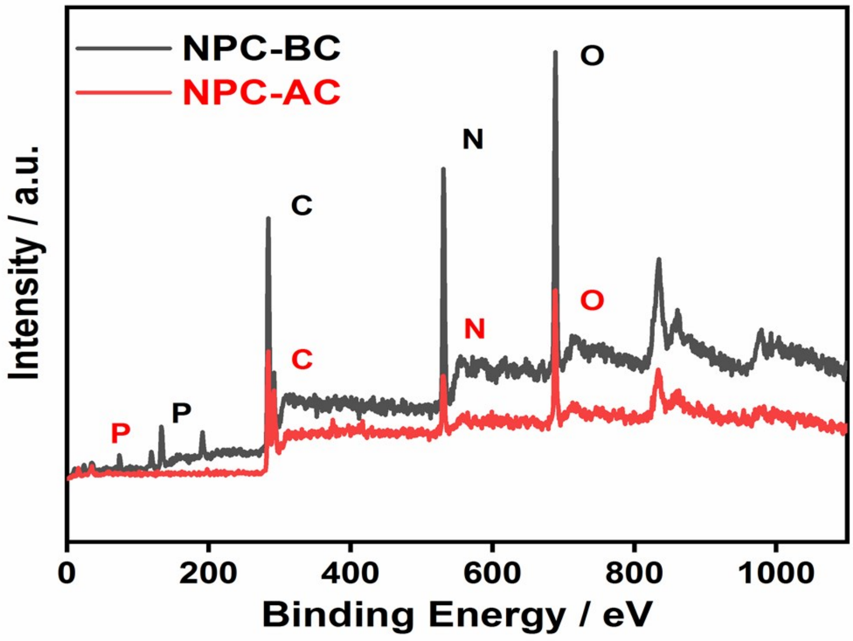

Figure S6. XPS (a) Survey spectrum of NPC before and after cycling.

Table S1. Various catalyst performance comparisons

**Electrocatalyst**	**Catalyst loading (mg/cm²)**	**Anode/ Electrolyte**	**Voltage gap**	**Charge-Discharge condition**	**Stability**	**Reference**
BSCF@Co-Nx-C	1 mg	Zn plate<br>6 M KOH + 0.2 M $Zn(AC)_2$	~0.83 V at 5 mA·cm^{-2}	5 mA·cm^{-2}<br>600 s per cycle period	1800 cycles; negligible voltage change at the end	1
FeNx-embedded PNC		Zn plate<br>0.5 M $(ZnSO_4)_2$	~0.78 V at 5 mA·cm^{-2}	5 mA·cm^{-2}, 660 s per cycle period	300 cycles; negligible voltage change at the end	2
FeCo-NCNFs-800		Zinc sheet<br>6 M KOH + 0.2 M $Zn(AC)_2$	~0.87 V at 10 mA cm^{-2}	10 mA cm^{-2}	Total time period- 2500 min; polarization increased ~0.11 V at the end	3
CMT@CNT	3	Zn foil,<br>6 M KOH + 0.2 M $Zn(AC)_2$	1.17 V @ j=20 mA cm^{-2}	20 mA cm^{-2}, 1200 s per cycle period	351 cycles; 117 hrs., polarization increased to 0.1 V at the end	4
$ZnCo_2O_4/Co_xSe_y$	4	Zn plate,<br>6 M KOH + 0.2 M $Zn(AC)_2.H_2O$	~0.82 V at 10 mA cm^{-2}	10 mA cm^{-2}, 600 s per cycle period	Total time period- 50 hr;<br>negligible voltage change at the end	5
NPC-"Li"		Zinc plate<br>6 M KOH + 0.2 M $Zn(AC)_2$	~1.22 V at 20 mA cm^{-2}	20 mA cm^{-2}, 116 s per cycle period	150 cycles; negligible voltage change at the end	6
$Ni_{1.5}Co_{1.5}S4$	1.5	Zinc plate<br>6 M KOH + 0.1 M $Zn(O_2CCH_3)_2$	~0.87 V @ j=4 mA cm^{-2}	j=4 mA cm^{-2},<br>28 s per cycle period	125 cycles; polarization increased 0.04 V at the end	7
$LaNiO_3$/N-CNT	0.72	Zn plate	~1.2 V @ j=20 mA	~17.6 mA cm^{-2}, 600 s per cycle	75 cycles; polarization	8

		6M KOH	cm^{-2}	period	increased 0.1–0.2 V at the end	
Co_3O_4-doped Co/CoFe	-	0.2 M zinc chloride + 6M KOH	0.9 V @ j= 5 mA cm^{-2}	5 mA cm^{-2}, 1300 s per cycle period	180 cycles; negligible voltage change at the end	9
a-MnO_2-$LaNiO_3$/CNTs			0.746 V @ j=20 mA cm−2	20 mA cm−2, 600s/cycle	75cycles; polarization increased ~0.11V at the end	10
CoO/N-CNT + NiFe LDH	1.0/5.0	Zn foil, 6 M KOH + 0.2 M $Zn(AC)_2$	~0.69 V @ j=10 mA cm^{-2}	10 mA cm^{-2}, 200 s per cycle period	60 cycles; negligible voltage change at the end	11
Nanostructured $NiCo_2O_4$	-	Zn plate 6 M KOH	0.70 V @ j=20 mA cm^{-2}	~20 mA cm^{-2}, 300 s per cycle period	50 cycles; polarization increased ~0.2 V at the end	12
$LaNiO_3$/rGO	10.1	Zn plate 6 M KOH	~0.8 V @ j=10 mA cm^{-2}; ~0.98 V @ j=25 mA cm^{-2}	~25 mA cm^{-2}, 3600 s per cycle period	97 cycles; negligible voltage change at the end	13
$NiCo_2S_4$/N-CNT	1.0	Zn foil 6 M KOH + 0.2 $ZnCl_2$	0.63 V @ j=10 mA cm^{-2}; 0.80 V @ j=20 mA cm^{-2}	10 mA cm^{-2}, 400 s per cycle period	150 cycles; polarization increased 0.06 V at the end	14
NPC	4mg	Stainless steel mesh 6 M KOH + ZnO	0.89 V @ j= 10 mA cm^{-2}	10 mA cm^{-2}, per cycle period 300 s	900 cycles; 100 hrs., polarization increased to 0.14 V at the end	This Work
NSC	4mg	Stainless steel mesh 6 M KOH + ZnO	0.89 V @ j= 10 mA cm^{-2}	10 mA cm^{-2}, per cycle period 300 s	600 cycles; 80 hrs., polarization increased to 0.14 V at the end	This Work

References

[1] Y. Arafat, M. R. Azhar, Y. Zhong, X. Xu, M. O. Tadé, Z. Shao, *Nano-Micro Lett*. 2020, **12**, 130.

[2] L. Ma, S. Chen, Z. Pei, Y. Huang, G. Liang, F. Mo, Q. Yang, J. Su, Y. Gao, J. A. Zapien and C. Zhi, *ACS Nano*. 2018, **12**, 2, 1949–1958

[3] L. Yang, S. Feng, G. Xu, B. Wei, and L. Zhang, *ACS Sustainable Chem. Eng.* 2019, **7**, 5, 5462–5475

[4] W. Xie, J. Li, Y.Song, S.Li, J. Li and M. Shao, *Nano-Micro Lett*. 2020,**12**, 97.

[5] Z. Mai, W. Duan, K. Wang, Z. Tang, and S. Chen, *Sustainable Energy Fuels*. 2020, **4**, 2184-2191

[6] P. Li, H. Jang, B. Yuan, Z. Wu, X. Liu and J. Cho, *Inorg. Chem. Front*. 2019, **6**, 417-422.

[7] Y. Xu, A. Sumboja, Y. Zong and J. A. Darr, *Catal. Sci. Technol*. 2020, **10**, 2173-2182.

[8] Z. Chen, A. Yu, D. Higgins, H. Li, H. Wang, and Z. Chen, *Nano Lett*. 2012, **12**, 4, 1946-1952.

[9] L. Tongtong, L. Yongxin, Z. Shuaishuai, G. Zhi-Da, S. Yan-Yan, *J. Mater. Chem. A.* 2018, **6**, 3730-3737.

[10] M. Hongyun, W. Baoguo, *RSC Adv.* 2014, **4**, 46084–46092.

[11] L. Yanguang, G. Ming, L. Yongye, J. Feng. K. Ji-Eun, W. Hailiang, H. Guosong, Z. Bo, D. Hongjie, *Nat. Commun*. 2013, **4**, 1805.

[12] M. Prabu, K. Ketpang and S. Shanmugam, *Nanoscale.* 2014, **6**, 3173-3181.

[13] C. Sheng, D. Jingjing, B. Pengju, T. Youhong, Z. Rongkun, Q. Shi-Zhang, *Adv. Energy Mater.* 2015, **5**, 1500936.

[14] H. Jie., L. Qiunan, S. Ziwei, L. Zhang, H. Huang, *RSC. Adv*. 2016, **6**, 86386-86394.
